# Sequence Polymorphism of Cytochrome b Gene in *Theileria annulata* Tunisian Isolates and Its Association with Buparvaquone Treatment Failure

**DOI:** 10.1371/journal.pone.0129678

**Published:** 2015-06-10

**Authors:** Moez Mhadhbi, Melek Chaouch, Kaouthar Ajroud, Mohamed Aziz Darghouth, Souha BenAbderrazak

**Affiliations:** 1 Laboratoire de Parasitologie, École Nationale de Médecine Vétérinaire, Université de la Manouba, 2020 Sidi Thabet, Tunisia; 2 Laboratoire de Parasitologie Médicale, Biotechnologies et Biomolécules, Institut Pasteur de Tunis, Tunis, Tunisia; 3 Faculté des Sciences de Bizerte, Université de Carthage, Tunis, Tunisia; Obihiro University of Agriculture and Veterinary Medicine, JAPAN

## Abstract

**Background:**

Buparvaquone (BW 720C) is the major hydroxynaphtoquinone active against tropical theileriosis (*Theileria annulata* infection). Previous studies showed that buparvaquone, similarly to others hydroxynaphtoquinone, probably acts by binding to cytochrome b (cyt b) inhibiting the electron transport chain in the parasite. Several observations suggested that *T*. *annulata *is becoming resistant to buparvaquone in many endemic regions (Tunisia, Turkey and Iran), which may hinder the development of bovine livestock in these areas.

**Methodology/Principal Findings:**

In the present study we sought to determine whether point mutations in *T*. *annulata* cytochrome b gene could be associated to buparvaquone resistance. A total of 28 clones were studied in this work, 19 of which were obtained from 3 resistant isolates (ST2/12, ST2/13 and ST2/19) collected at different time after treatment, from a field treatment failure and nine clones isolated from 4 sensitive stocks of *T*. *annulata *(Beja, Battan, Jed4 and Sousse). The cytochrome b gene was amplified and sequenced. We identified five point mutations at the protein sequences (114, 129, 253, 262 and 347) specific for the clones isolated from resistant stocks. Two of them affecting 68% (13/19) of resistant clones, are present in the drug-binding site Q_02_ region at the position 253 in three resistant clones and at the position 262 in 11 out of 19 resistant clones. These two mutations substitute a neutral and hydrophobic amino acids by polar and hydrophilic ones which could interfere with the drug binding capabilities. When we compared our sequences to the Iranian ones, the phylogenetic tree analyses show the presence of a geographical sub-structuring in the population of *T*. *annulata*.

**Conclusions/Significance:**

Taken together, our results suggest that the cytochrome b gene may be used as a tool to discriminate between different *T*. *annulata* genotypes and also as a genetic marker to characterize resistant isolates of *T*. *annulata*.

## Introduction

Tropical theileriosis is a tick-borne disease caused by the protozoan parasite *Theileria annulata*. It affects cattle belonging to a large geographical area that includes southern Europe, North Africa and Asia. The high susceptibility of exotic breeds and crossbred cattle to the disease causes substantial financial losses and constitutes a major obstacle to the development of animal wealth [1]. Indeed, in the absence of treatment, the lethality of exotic breeds can reach 40–50% [2].

Since four decades, hydroxynaphtoquinones were found to have a strong theilericidal activity [3]. This discovery led to the development of parvaquone (BW 993C) [4] and subsequently buparvaquone (trans-2-[4-(4-butylcyclohexylmethyl)]-3-hydroxy-1,4-naphtoquinone) (BW 720C) [5] which shows a low toxicity and is high efficiency against *Theileria* infections in cattle. This latter compound is widely used to date in endemic regions for the treatment of clinical cases due to pathogenic *Theileria* species [6]. In Tunisia, buparvaquone was introduced in 1988 and has been successfully used in the field as it reduced the lethality to less than12% [7].

Since 1996, Tunisian field veterinarians reported unusual failures in the treatment of clinical cases. Indeed, despite an early treatment with a conventional dose (2.5 mg/kg), these cases result in either a fatal outcome or a survival of the cattle with however a persistent high level of parasiteamia and a small number of degenerative forms of *T*. *annulata*. We have experimentally confirmed, *in vivo*, the emergence of *T*. *annulata* resistant strains in Tunisia [8].

The mechanism of buparvaquone action has not been fully elucidated. However, products belonging to the hydroxynaphtoquinones family such as parvaquone and other 1,4-naphtoquinone, competitively inhibit the binding of ubiquinone (coenzyme Q) to cytochrome b of the mitochondrial cytochrome bc1 complex [9]. This results in the inhibition of electron transport and the collapse of mitochondrial membrane potential. Similar mechanism have been demonstrated for atovaquone (trans-2-[4-(4-chlorophenyl) cyclohexyl]-3-hydroxy-1,4-naphthalenedione), another hydroxynaphtoquinone used to control *Plasmodium* spp., *Toxoplasma gondii* and *Pneumocystis carinii* infections [10].

On the other hands, resistance to atovaquone revealed by treatment failure has been reported for *Plasmodium falciparum* [11], *P*. *carinii* [12] and *T*. *gondii* [13]. The analysis of these resistant parasites has associated the resistance to mutations in the cyt b ubiquinone binding site [14–15]. The mechanism of *T*. *annulata* resistance to buparvaquone might be caused by a similar mechanism i.e. mutations in the binding site of ubiquinone, since buparvaquone may also acts by binding to cyt b.

We report, in the present study, a molecular analysis of cyt b gene of *T*. *annulata* resistant and susceptible to buparvaquone clones. Our results point to different specific mutations in the cyt b gene that affect the binding site of ubiquinone, in the *T*. *annulata* clones obtained from resistant field case[8].

## Material and Methods

### Biological material description

This study was carried out using four sensitive and three resistant*T*. *annulata* stocks. The four *T*. *annulata* stocks, Beja, Jed4, Battan and Sousse, susceptible to conventional treatment with buparvaquone, were isolated from different Tunisian endemic regions [16]. These stocks, have been respectively isolated from North western, North eastern and east central of Tunisia. Three resistant stocks of *T*. *annulata*, namely ST2/12, ST2/13 and ST2/19, were isolated at various times after treatment from a well documented tropical theileriosis clinical case of buparvaquone treatment failure. This case (cow 7) was identified in a farm located in the delegation of Sidi Thabet (ST), North eastern Tunisia [8]. The cow recovered long time after the three intramuscular injections of buparvaquone (at the conventional dose of 2.5 mg/kg), with the first two administered at an interval of 72 hours and the third injected 48h later. However, this cow continued to exhibit a consistent heamatocrit decrease and a high parasiteamia. We characterized the resistance of stocks on the absence of the usual effect of buparvaquone such a rapid decline of parasiteamia and a recovery time that do not exceed within 3 days [4]. The first (ST2/12) and the second isolates (ST2/13) were respectively sampled the day of the first treatment and 24 hours later, while, the last sample (ST2/19), was taken 48 hours after the third treatment. From these 7 stocks kept during a maximum of 3 days in cell culture (to avoid the *in vitro* pressure selection), 28 clones were generated using limiting dilution [17] (Table 1).

**Table 1:** characterization of *Theileria annulata* isolates used in the study.

Buparvaquone activity	Isolate	Sampling date	Clone
**Susceptible stocks**	Beja		BejaC2, BejaC3
	Sousse		SousseC2
	Battan		BattanC1, BattanC2, BattanC3, BattanC4, BattanC5
	Jed4		Jed4C2
**Resistant stocks**	ST2/12	The day of the first treatment	ST2/12C3, ST2/12C4, ST2/12C5, ST2/12C6
	ST2/13	24 hours after the first treatment	ST2/13C2, ST2/13C6, ST2/13C7, ST2/13C13
	ST2/19	48 hours after the third treatment	ST2/19C1, ST2/19C3, ST2/19C4, ST2/19C5, ST2/19C6, ST2/19C10, ST2/19C11, ST2/19C13, ST2/19C16, ST2/19C18, ST2/19C19

### DNA extraction and PCR conditions

The cell lines stocks cryopreserved in liquid nitrogen, were thawed and cultured in 25 cm^2^ tissue culture flasks using RPMI 1640 medium (Gibco/Invitrogen, USA), supplemented with 10% foetal calf serum (Gibco/Invitrogen, USA). Approximately 10^7^ infected cells were centrifuged at 1500 g during 5 minutes and the cell pellet was washed in phosphate buffered saline (PBS). The final cell pellet was re-suspended in PBS and DNA was extracted using a Promega Wizard Genomic DNA Purification kit (Madison, WI, USA) according to the manufacturer’s instructions.

Extracted DNA was eluted in 100 μl of “Ultra Pure DNase/RNase-Free Distilled Water” (Gibco/Invitrogen, USA). Degenerated primers designed from the reference sequence of GenBank accession number XM949625 (*T*. *annulata*, strain Ankara), cytoF (5’ CAGGGCTTTAACCTACAAATTAAC3’) and cytoR (5’ CCCCTCCACTAAGCGTCTTTCGACAC 3’) were used to amplify a 1092 bp of cyt b gene. The PCR reactions were conducted in 50 μl final volume containing 2 μl of purified DNA sample, 1.5 mM MgCl2, 0.2 μM of each deoxynucleotide, 2.5U of Taq DNA polymerase (GE Healthcare, USA) and 60 pmol of each primer. Reactions were carried out in a Applied Biosystem, GeneAmp PCR System 2700 using the following cycling conditions: an initial denaturation step at 94°C for 5 min, 30 cycles at 94°C for 1min, 54°C for 1 min and 72°C for 1 min and a final elongation step at72°C for 10 min. Amplicons were electrophoresed in 1% agarose gels stained with ethidium bromide and visualized under UV light.

### DNA sequencing

The DNA fragments obtained after PCR assays were purified using the QIAquick PCR purification kit (QIAGEN) according to the supplier’s instructions. Then, the DNA sequence was determined using a conventional Big Dye Terminator cycle sequencing ready reaction kit (Perkin Elmer, Applied Biosystems, Foster City, CA) and an ABI373 Automated DNA Sequence. The comparison of the sequences was made with the reference sequence XM949625 of *T*. *annulata* cyt b gene using BLAST (Basic Local Alignment Search Tool). The DNA and predicted amino acid sequences were aligned and compared using the Clustal W program [18]. The Clustal output was edited and the region of unambiguously aligned sequences was retained for final analysis. Phylogenetic analyses based on a protein sequence of 217 amino acids were carried out using the MEGA 5 software.

## Results

### Cytochrome b sequence analyses

The 1092 bp nucleotide sequences corresponding to the complete cyt b gene sequence were amplified and sequenced. The sequences of the 28 Tunisians clones were compared with the other related cyt b gene sequences in the GenBank database.

Nine novel sequences were identified. All data were deposited into GeneBank data base under the following accession numbers: KF732022, KF732023, KF732024, KF732025, KF732026, KF732027, KF732028, KF732029 and KF732030.

The alignment of the identified nucleotide sequences with the reference *T*. *annulata* cyt b gene (XM949625) showed a specific polymorphism at seventeen positions in the Tunisian clones. The alignment of our amino acid sequences with the reference show that ten among these are non-synonymous mutations, which cause a change in the polypeptide chain of cyt b (Table 2).

**Table 2:** Mutations registered in the cytochrome b gene of the Tunisian clones.

Nucleotide		30	61	138	342	385	417	427–429	436	445	593	655	757	785	849	870	1040
**Codon**		10	21*	46	114*	129*	139	143	146*	149	198*	219*	253*	262*	283	290	347*
	Tancytb (XM949625)	TCG	TGG	GTG	ATA	AGC	TTA	TTC	GCT	CTA	ATA	GTT	CCT	TTA	GCA	GTA	TCA
**Sensitive clones**	BattanC1	…	…	..**A**	…	…	..**G**	..**T**	**A**..	…	**G**..	**A**..	…	…	…	..**G**	…
	BattanC2	…	…	..**A**	…	…	..**G**	..**T**	**A**..	..	**G**..	**A**..	…	..	…	..**G**	…
	BattanC3	…	…	..**A**	…	…	..**G**	..**T**	**A**..	…	**G**..	**A**..	…	…	…	..**G**	…
	BattanC4	…	…	..**A**	…	…	..**G**	..**T**	**A**..	…	**G**..	**A**..	…	…	…	..**G**	…
	BattanC5	…	…	..**A**	…	…	..**G**	..**T**	**A**..	…	**G**..	**A**..	…	…	…	..**G**	…
	BejaC2	…	…	..**A**	…	…	..**G**	..**T**	**A**..	…	…	**A**..	…	…	..**G**	..**G**	…
	BejaC3	…	…	..**A**	…	…	..**G**	..**T**	**A**..	…	…	**A**..	…	…	..**G**	..**G**	…
	Jed4C2	…	**C**..	..**A**	…	…	..**G**	* **C**.**T**	**A**..	**T**..	…	**A**..	…	…	…	..**G**	…
	SousseC2	…	…	..**A**	…	…	..**G**	..**T**	**A**..	…	…	**A**..	…	…	..**G**	..**G**	…
**Resistant clones**	ST2/12C3	…	…	..**A**	…	…	..**G**	..**T**	**A**..	…	…	**A**..	**T**..	…	…	..**G**	…
	ST2/12C4	..**A**	…	..**A**	…	…	..**G**	..**T**	**A**..	…	…	**A**..	…	…	…	..**G**	.**T**.
	ST2/12C5	…	…	..**A**	..**G**	…	..**G**	..**T**	**A**..	…	…	**A**..	…	…	…	..**G**	…
	ST2/12C6	…	…	..**A**	…	**G**..	..**G**	..**T**	**A**..	…	…	**A**..	…	…	…	..**G**	…
	ST2/13C2	…	…	..**A**	…	…	..**G**	..**T**	**A**..	…	…	**A**..	…	.**C**.	…	..**G**	…
	ST2/13C6	..**A**	…	..**A**	…	…	..**G**	..**T**	**A**..	…	…	**A**..	…	…	…	..**G**	.**T**.
	ST2/13C7	…	…	..**A**	…	…	..**G**	..**T**	**A**..	…	…	**A**..	…	.**C**.	…	..**G**	…
	ST2/13C13	…	…	..**A**	…	**G**..	..**G**	..**T**	**A**..	…	…	**A**..	…	…	..**G**	..**G**	…
	ST2/19C1	…	…	..**A**	…	…	..**G**	..**T**	**A**..	…	…	**A**..	…	.**C**.	…	..**G**	…
	ST2/19C3	..**A**	…	..**A**	…	…	..**G**	..**T**	**A**..	…	…	**A**..	…	…	…	..**G**	.**T**.
	ST2/19C4	…	…	..**A**	…	…	..**G**	..**T**	**A**..	…	…	**A**..	…	.**C**.	…	..**G**	…
	ST2/19C5	…	…	..**A**	…	…	..**G**	..**T**	**A**..	…	…	**A**..	…	.**C**.	…	..**G**	…
	ST2/19C6	…	…	..**A**	…	…	..**G**	..**T**	**A**..	…	…	**A**..	…	.**C**.	…	..**G**	…
	ST2/19C10	…	…	..**A**	…	…	..**G**	..**T**	**A**..	…	…	**A**..	**T**..	.**C**.	…	..**G**	…
	ST2/19C11	…	…	..**A**	…	…	..**G**	..**T**	**A**..	…	…	**A**..	…	.**C**.	…	..**G**	…
	ST2/19C13	…	…	..**A**	…	…	..**G**	..**T**	**A**..	…	…	**A**..	…	.**C**.	…	..**G**	…
	ST2/19C16	…	…	..**A**	…	…	..**G**	..**T**	**A**..	…	…	**A**..	**T**..	…	…	..**G**	…
	ST2/19C18	…	…	..**A**	…	…	..**G**	..**T**	**A**..	…	…	**A**..	…	.**C**.	…	..**G**	…
	ST2/19C19	…	…	..**A**	…	…	..**G**	..**T**	**A**..	…	…	**A**..	…	.**C**.	…	..**G**	…

*: Nonsynonymous mutation

The clones isolated from the sensitive stocks showed 4 specific mutations. Indeed, Jed4C2 (KF732023) revealed two nonsynonymous mutations at the codon 21 and 143, (with a double point mutation in codon 143 (TCT/CTT)) and one silent mutation at the codon 149. The five clones isolated from the stock Battan (KF732022) revealed one nonsynonymous mutation (198). Clone BejaC2, BejaC3 and SousseC2 showed one specific silent mutation at the codon 283.

Five nonsynonymous mutations (114, 129, 253,262 and 347) and one synonymous one (10) were exclusively detected in the resistant clones. Hence, the KF732027 sequence characterized by a specific nonsynonymous mutation at the codon 129 was found in two resistant clones ST2/12C6 and ST2/13C13. The KF732025 sequence that reveals a silent mutation at codon 347 was found in three other resistant clones (ST2/12C4, ST2/13C6 and ST2/19C3). The mutation at the position (114) was detected in one resistant clone (KF732026). The mutation (253) was present in three resistant clones (KF732024 and KF732030). The mutation (262) was detected in 11 out of the 19 resistant clones (KF732028 and KF732030) (Table 3). These results suggest a marked polymorphism between susceptible and those resistant clones but also between the resistant clones themselves. In fact, we must point out that (i): all the resistant clones (4) isolated the day of the first treatment (ST2/12) show one of the nonsynonymous mutations 114, 129, 253 or 347 absent in the sensitive clones. (ii): the number of clone, with specific nonsynonymous mutations, rises as the number of treatment increases.

**Table 3:** Specific nucleotide substitutions in cytochrome b gene from sensitive and resistant *Theileria annulata* clones.

GenBank accession number		Nucleotide	61	342	385	427	593	757	785	1040
		Codon	21	114	129	143	198	253	262	347
XM949625	Tancytb		**T**GG (*Trp*)	**A**TA (*Ile*)	**A**GC(*Ser*)	**T**TC(*Phe*)	**A**TA(*Ile*)	**C**CT(*Pro*)	**T**TA(*Leu*)	T**C**A(*Ser*)
KF732029	BejaC2, BejaC3, SousseC2									
KF732022	BattanC1, BattanC2, BattanC3, BattanC4, BattanC5						**G**TA(*Val*)			
KF732023	Jed4C2		**C**GG(*Arg*)			**C**TT(*Leu*)				
KF732024	ST2/12C3, ST2/19C16							**T**CT(*Ser*)		
KF732025	ST2/12C4, ST2/13C6, ST2/19C3									T**T**A(*Leu*)
KF732026	ST2/12C5			AT**G** (*Met*)						
KF732027	ST2/12C6, ST2/13C13				**G**GC(*Gly*)					
KF732028	ST2/13C2, ST2/13C7,									
	ST2/19C1, ST2/19C4									
	ST2/19C5, ST2/19C6								T**C**A(*Ser*)	
	ST2/19C11, ST2/19C13									
	ST2/19C18, ST2/19C19									
KF732030	ST2/19C10							**T**CT(*Ser*)	T**C**A(*Ser*)	
						**Q_01_**		**Q_02_**		

We next compared our sequences to those of three Iranian ones (JQ308837, JQ308838 and JQ308839 incomplete cyt b gene sequences (651 bp)). Compared to the reference sequence, these show three silent mutations (codon: 139, 143 and 290) found in all the Tunisian sequences.

### Phylogenetic analysis

**Fig 1:**
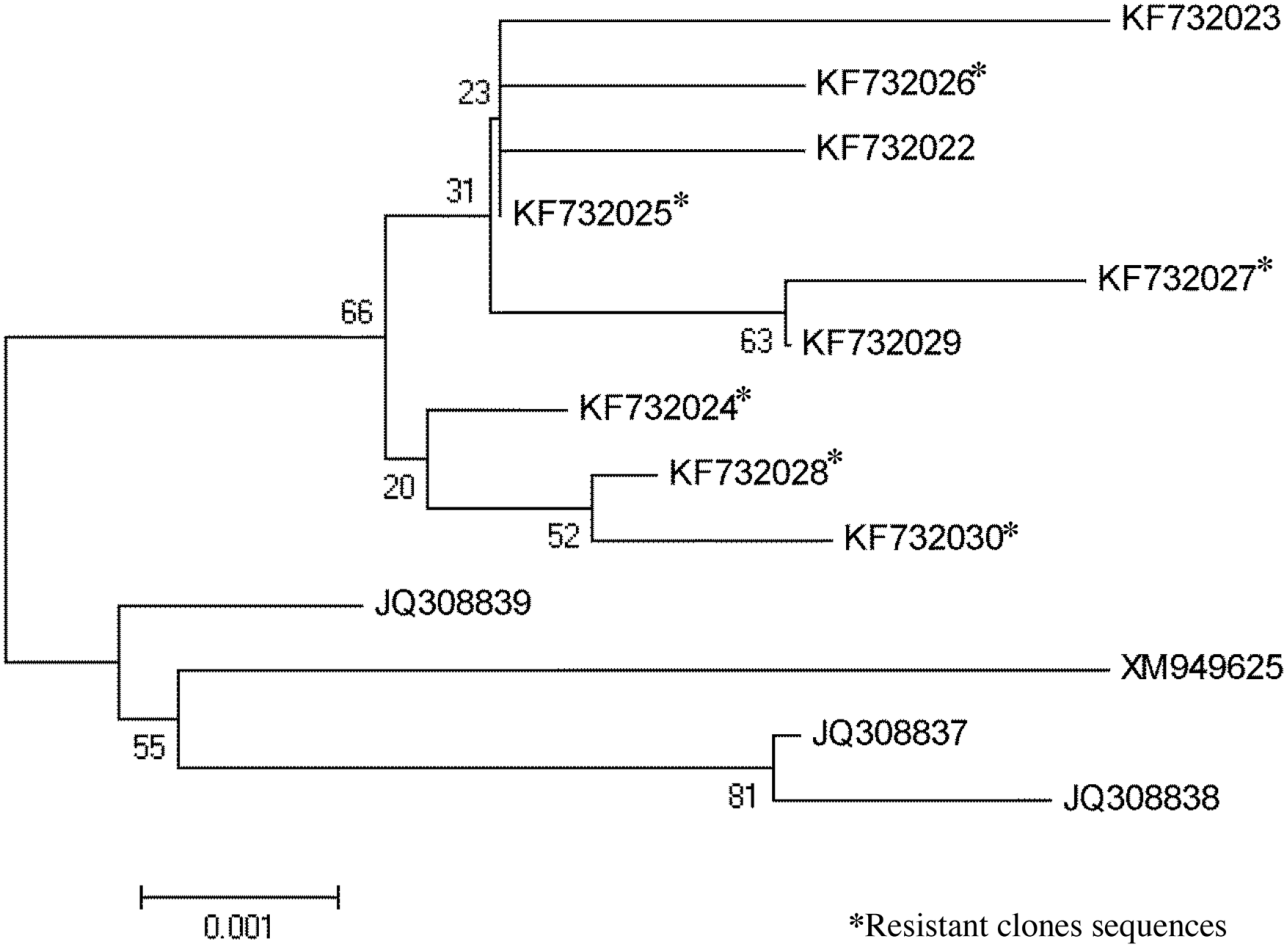
Phylogenetic tree based on the cytochrome b gene of *T*. *annulata*. Tunisian clones sequences. Phylogenetic tree generated using the neighbour-joining algorithm based on the sequence alignment of the cytochrome b gene of *T*. *annulata*. Tunisian clone sequences: KF732029, KF732022, KF732023, KF732026, KF732025, KF732027, KF732024, KF732028, KF732030; Iranian isolates: JQ308839, JQ308838, JQ308837; Ankara strain (Turkey) XM949625. Bootstrap values are shown as percentages at each node based on 1000 replicates.

A phylogenetic tree based on the edited alignment of the cyt b protein sequences (217 amino acids) of Tunisian clones, Iranian isolates and the Ankara (reference) isolate is generated using the neighbor joining method. It divides the full studied strains into two clusters (Fig 1). One cluster that includes the reference and the Iranian sequences and a second cluster that holds two nodes dividing the *T*. *annulata* Tunisian sequences into two groups. The first group of sequences is composed by the nine clones isolated from sensitive stocks (KF732029, KF732022 and KF732023) and six clones isolated from the resistant stock (KF732025, KF732026 and KF732027). The second group is composed exclusively by the Tunisian resistant clones (KF732024, KF732028 and KF732030), which present specific mutations (253 and 262).

### Mutations associated with *Theileria annulata* buparvaquone resistance

The alignment of amino acid sequences of all Tunisian clones was performed with Clustal W algorithm. We first analyzed the Q_01_ (130–148) and Q_02_ (244–266) regions in the drug binding site. Our results revealed the presence of one mutation (146) affecting the Q_01_ region of all the studied clones, with clone Jed4C2 (KF732023), presenting in addition a double nucleotide substitution in the codon 143 which results in amino acid substitution from phenylalanine to leucine. None of the resistant clones show specific mutations in the Q_01_ region. However, one nonsynonymous mutation was registered close to the Q_01_ region at codon 129 in two resistant clones (KF732027). In the Q_02_ region, no mutations were found in the sensitive clones, whereas, two mutations were noticed in resistant clones. These changes resulted in amino acid substitution from proline (253) to serine for three resistant clones (KF732024 and KF732030) and from leucine (262) to serine in 11 resistant clones (KF732028 and KF732030), which correspond to a prevalence of 15% (3/19) for the former and 58% for the latter (11/19) (Table 3).

Analyzing the frequency of mutations in relation to the moment of isolation (after treatment) we found that the four clones isolated from ST2/12 (sampled the day of the first treatment), presented different nonsynonymous mutations (*Ile114Met*, *Ser129Gly*, *Pro253Ser* and *Ser347Leu*). Among the four clones isolated from ST2/13 (sampled 24 hours after the first treatment), two clones (ST2/13C2 and ST2/13C7) showed the same mutation (*Leu262Ser*) and the two others (ST2/13C13 and ST2/13C6) showed two different mutations (*Ser129Gly* and *Ser347Leu* respectively). By contrast, 9 out of 11 (81%) clones isolated from ST2/19 (sampled 48 hours after the third treatment) showed the same mutation (*Leu262Ser*) in the Q_02_ region, while a single clone (ST2/19C10) presented a double mutation (*Pro253Ser* and *Leu262Ser*) and one clone (ST2/19C16) showed a *Pro253Ser* mutation.

In conclusion, all resistant clones revealed a mutation in the cyt b gene. The most frequent mutation was *Leu262Ser*, which affects the Q_02_ region, and represent 58% (11/19) of resistant clones but also 82% (9/11) of clones isolated 48 hours after the third treatment.

## Discussion

The occurrence of *T*. *annulata* resistance to buparvaquone, member of hydroxynaphtoquinones, has been described in Tunisia since 1996 by Darghouth et al. (unpublished data). These cases have been detected in a farm where four out of seven cows have succumbed to tropical theileriosis despite early treatment with the conventional doses of buparvaquone. *In vivo* experimental study confirmed the resistance of *T*. *annulata* isolates to buparvaquone [8]. In an attempt to collect parasites exposed to different degrees of drug pressure and thus to increase the probability to select resistant strains, we sampled a case of treatment failure at different times after treatment. The isolates where next cloned by limiting dilution. This allowed us to recover over 40 clones from three resistant isolates and 14 clones from four sensitive stocks (data not shown). The isolate, ST2/19 (collected after the third treatment) gave a larger number of clones (21) while ST2/12 and ST2/13 (collected after the first treatment) gave respectively 10 and 12 clones. The large number of clones derived from the resistant isolates suggests a relationship between resistance and adaptation to multiplication under cell culture conditions. This observation must however, be confirmed by a larger number of clones and statistical studies.

Naphtoquinones has been widely used against several protozoa, such as *Plasmodium* spp. [19], *Leishmania* spp. [20] and *P*. *carinii* [21]. It binds to the pathogens’ cytochrome bc1 complex [22]. Different studies have shown the association between the resistance to hydroxynaphtoquinones and the mutation in the cyt b catalytic site, the oxidation quinol site, namely coenzyme Q_0_. A previous study have defined two putative regions, namely Q_01_ and Q_02_, as a drug-binding site in *P*. *falciparum* and showed that mutations in these regions confer resistance of *P*. *falciparum* to atovaquone [23]. These regions were localized respectively between the codons 130–148 and 244–266.

We show here that resistance of *T*. *annulata* to buparvaquone is also associated to mutations in the Q_0_ cytb gene region. Our result is in accordance with those reported by others [24–25]. These studies have showed single mutations in the cytochrome b gene in the samples of *T annulata* isolated from clinical cases of treatment failure. Indeed, the analysis of cyt b gene sequences showed mutations in the functional drug binding regions Q_0_. The mutations, identified in the Iranian study [24] were detected in eight *T*. *annulata* isolates and have been reported to result in the substitution of two amino acids. The study conducted in Turkey [25], showed the presence of two other nonsynonymous mutations located at position 135 and 253 that resulted in amino acid substitution from valine to alanine and from proline to serine respectively. These studies have relied on the molecular analysis of isolates collected from the clinical cases of resistance of tropical theileriosis. We have in this study, used *T*. *annulata* resistant strains experimentally confirmed *in vivo*. Moreover, the multiclonal character of *T*. *annulata* isolates collected from such clinical cases [26–27], prompted us to clone the isolates by limiting dilution which allowed us to analyse a greater number of sequences and to identify a larger number of mutation.

Compared to the of *T*. *annulata* cyt b gene reference (XM949625) we detected ten nonsynonymous mutations in the studied clones (19 resistant and 9 sensitive), five of them being not present in the sensitive clones.

None of the resistant clone has a specific mutation in the Q_01_ domain, which suggests that mutations’ affecting this domain does not appear to be involved in resistance. The unique nonsynonymous mutation (*Ala146Thr*) affecting this region in the resistant clones, was also recorded in all Tunisian clones. Moreover, the only specific mutation affecting the Q_01_ region was found in the sensitive clone Jed4C2 (*Phe143Leu*). Mutations outside but close to the Q_01_ and Q_02_ regions (*Ser129Gly* and *Ser347Leu*), found only in the resistant clones (KF732027 and KF732025 respectively), may also play a role in the drug resistance as mutations described for *Pneumocystis jirovecii* [28].

The mutations *Pro253Ser* and *Leu262Ser* occurring respectively, in 16% (3/19) and 58% (11/19) of the resistant clones, were located in the Q_02_ region (Table 3). One of these, mutation *Pro253Ser*, was described in the Turkey study [25] with a prevalence of 8.3%. This result is illustrated by the phylogenetic tree analyses that show two clusters (Fig 1): a cluster that includes the reference and the Iranian sequences and a second cluster that includes all the *T*. *annulata* Tunisian sequences. This latter also shows two nodes dividing the *T*. *annulata* Tunisian sequences into two groups. One of those includes exclusively the resistant clones with mutations located in the Q_02_ region. The second node includes sensitive and resistant clones missing mutations in the Q_0_ regions. Taken together, these results suggest the presence of a geographical sub-structuring in the population of *T*. *annulata* [29], it also demonstrate that the cytochrome b gene can be used as a tool to discriminates between different *T*. *annulata* genotypes [30] and also as a genetic marker to characterize resistant isolates of *T*. *annulata*.

It’s important to point out that these two mutations (*Pro253Ser* and *Leu262Ser*) which affect 68% (13/19) of resistant clones,substitute an hydrophobic amino acid (leucine and proline) by an hydrophilic one (serine). This change in the hydrophobicity could explain the decreased affinity of buparvaquone to cytochrome b. Indeed, it was shown that 12 out of the 17 amino acids that link the cyt b protein to atovaquone are hydrophobic for *P*. *falciparum* [26]. Indeed, the hydrophobicity of the link between atovaquone and cyt b has been previously confirmed [31]. On the other hand, *in silico* model confirms that such mutations, by inducing a conformational change and a reduction in the volume of the-binding pocket interfere with the binding capacity of the drug [28–32]. The destabilization of the hydrophobic/aromatic interaction was found to be another effect of the cyt b gene mutations in *P*. *falciparum* [31].

However, the mutation *Pro253Ser* with the mutation *Leu262Ser* were detected respectively in the clone ST2/12C3 and ST2/13C3 and ST2/13C7 derived from the first samples and in those issued from ST2/19 collected after the third treatment. Furthermore, the mutation *Ser347Leu* was detected in different clones derived from each of the three isolates (Table 3). We can thus assume that the cloning procedure is not behind the emergence of resistant clones. Many assumptions have been made to explain the high frequency of spontaneous mutations in the mitochondrial genome including the less efficient proofreading compared with the nucleus [14–33], the multi-copy type of the mitochondrial cyt b gene [34] and the promotion of the mitochondrial DNA mutagenesis due to the hydroxyl radicals generated by the mitochondrial respiration chain [35].

In the field, the resistance could be due to the use of low buparvaquone dose and the pressure of selection generated by the massive use of buparvaquone during more than thirty years.

Tropical theileriosis is a major obstacle to the development of cattle breeding in Tunisia. The emergence and dissemination of resistant strains of *T*. *annulata* to buparvaquone will further aggravate the situation. It will be certainly of great interest to develop molecular markers and phenotypic tests to detect occurrence of resistance and determine its prevalence in the population and model its evolution spread.

The mechanism of resistance needs further investigation to determine whether mutations in the cytb are behind the resistance of *T*. *annulata* to buparvaquone.
